# *miR-124* dosage regulates prefrontal cortex function by dopaminergic modulation

**DOI:** 10.1038/s41598-019-38910-2

**Published:** 2019-03-05

**Authors:** Takashi Kozuka, Yoshihiro Omori, Satoshi Watanabe, Etsuko Tarusawa, Haruka Yamamoto, Taro Chaya, Mayu Furuhashi, Makiko Morita, Tetsuya Sato, Shinichi Hirose, Yasuyuki Ohkawa, Yumiko Yoshimura, Takatoshi Hikida, Takahisa Furukawa

**Affiliations:** 1 Laboratory for Molecular and Developmental Biology, Institute for Protein Research, Osaka University, 3-2 Yamadaoka, Suita, Osaka 565-0871 Japan; 2 JST, CREST, 3-2 Yamadaoka, Suita, Osaka 565-0871 Japan; 3 Division of Visual Information Processing, National Institute for Physiological Sciences, National Institutes of Natural Sciences, 38 Nishigonaka Myodaiji, Okazaki, 444-8585 Japan; 4 Medical Innovation Center, Kyoto University Graduate School of Medicine, 53 Shogoin-Kawahara-cho, Sakyo-ku, Kyoto 606-8507 Japan; 5 Division of Bioinformatics, Medical Institute of Bioregulation, Kyushu University, 3-1-1 Maidashi, Higashi-ku, Fukuoka 812-8582 Japan; 6 Division of Transcriptomics, Medical Institute of Bioregulation, Kyushu University, 3-1-1 Maidashi, Higashi-ku, Fukuoka 812-8582 Japan; 7 Department of Pediatrics, School of Medicine, Fukuoka University, 45-1-7 Nanakuma, Jonan-ku, Fukuoka 814-0180 Japan; 8 Laboratory for Advanced Brain Functions, Institute for Protein Research, Osaka University, 3-2 Yamadaoka, Suita, Osaka 565-0871 Japan

## Abstract

*MicroRNA-124* (*miR-124*) is evolutionarily highly conserved among species and one of the most abundantly expressed miRNAs in the developing and mature central nervous system (CNS). Previous studies reported that *miR-124* plays a role in CNS development, such as neuronal differentiation, maturation, and survival. However, the role of *miR-124* in normal brain function has not yet been revealed. Here, we subjected *miR-124-1*^+/−^ mice, to a comprehensive behavioral battery. We found that *miR-124-1*^+/−^ mice showed impaired prepulse inhibition (PPI), methamphetamine-induced hyperactivity, and social deficits. Whole cell recordings using prefrontal cortex (PFC) slices showed enhanced synaptic transmission in layer 5 pyramidal cells in the *miR-124-1*^+/−^ PFC. Based on the results of behavioral and electrophysiological analysis, we focused on genes involved in the dopaminergic system and identified a significant increase of *Drd2* expression level in the *miR-124-1*^+/−^ PFC. Overexpression or knockdown of *Drd2* in the control or *miR-124-1*^+/−^ PFC demonstrates that aberrant *Drd2* signaling leads to impaired PPI. Furthermore, we identified that expression of glucocorticoid receptor gene *Nr3c1*, which enhances *Drd2* expression, increased in the *miR-124-1*^+/−^ PFC. Taken together, the current study suggests that *miR-124* dosage modulates PFC function through repressing the *Drd2* pathway, suggesting a critical role of *miR-124* in normal PFC function.

## Introduction

MicroRNAs (miRNAs) are small non-coding RNA molecules regulating gene expression of a great variety of biological processes in plants and animals. A large number of diverse miRNAs are expressed in the vertebrate CNS. *MicroRNA-124* (*miR-124*) is one of the most abundantly expressed miRNAs in mouse and human brains^1^. The nucleotide sequence of *miR-124* and its nervous system-specific expression pattern are highly evolutionarily conserved from *C. elegans* through *Drosophila melanogaster*, and all vertebrates studied through to humans. Both in human and mouse genomes, *miR-124s* are encoded on three loci: *miR-124-1*, *-2*, and *-3*. Among the three primary *miR-124s* (*pri-miR-124s*), *pri-miR-124-1* is predominantly expressed^2^. In mice, *retinal non-coding RNA3* (*RNCR3*) functions as a *pri-miR-124-1* precursor. We previously identified *RNCR3* in the mouse retina and generated *RNCR3*-deficient mice (*RNCR3*^−/−^) by deleting the entire 4.5 kb region harboring *RNCR3*^2^. In the *RNCR3*^−/−^ brain, the level of mature *miR-124* was reduced by 60–80% compared with that in the wild-type (WT) brain. *RNCR3*^−/−^ mice showed abnormalities in the CNS such as over-extension of dentate gyrus granule neuron axons and apoptosis of cone photoreceptor cells in the retina^2^. Since in our previous study we confirmed that a loss of *miR-124* is responsible for the *RNCR3*^−/−^ abnormalities by *in vivo* rescue experiment, we will refer to *RNCR3*^−/−^ mice as *miR-124-1*^−/−^ mice in the current study.

In *C. elegans miR-124* mutants, neurons are normally generated and no overt phenotype was observed^3,4^. *Drosophila miR-124* mutants exhibit abnormalities in neuroblast proliferation and neuronal maturation^5,6^. In vertebrates, consistent with its expression pattern in the developing CNS, *miR-124* has been reported to be essential for neuronal differentiation^7,8^, maturation^2,9–11^, synaptic plasticity^12,13^ and progenitor proliferation^7^, however, it should be noted that conflicting results on *miR-124* function have been reported. It was reported that neither inhibition nor overexpression of *miR-124* affected neuronal differentiation or progenitor proliferation in the chick neural tube^9^. On the other hand, other studies reported that *miR-124* promotes both embryonic and adult neurogenesis^7,14^. In the chick spinal cord, *miR-124* was implicated in the stimulation of neuronal differentiation through suppressing the anti-neuronal REST/SCP1 pathway. In addition, *miR-124* induces neurogenesis in P19 mouse embryonic cells^7^. In the adult mouse subventricular zone, *miR-124* was shown to induce adult neurogenesis through regulation of *Sox9*^14^.

Human *miR-124-1* is located in the chromosome 8p23.1 region. Heterozygous deletions on human 8p23.1 have been reported to be associated with psychiatric disorders, including schizophrenia, autism, and social impairment^15–18^. Due to its location on 8p23.1^19^ and because of its specific expression to the CNS, human *miR-124-1* is implicated to be associated with mental diseases.

Despite of many studies, the role of *miR-124* in normal brain function remains unclarified. While *miR-124-1*^−/−^ mice show aberrant growth of dentate granule cell axons in the hippocampus^2^, *miR-124-1*^+/−^ mice exhibited no substantial morphological defects in the brain development as far as we analyzed. In the current study, we investigated the role and mechanism of *miR-124* in normal brain functions using *miR-124-1*^+/−^ mice.

## Results

### A comprehensive behavioral test battery for *miR-124-1*^+/−^ mice

**Figure 1:**
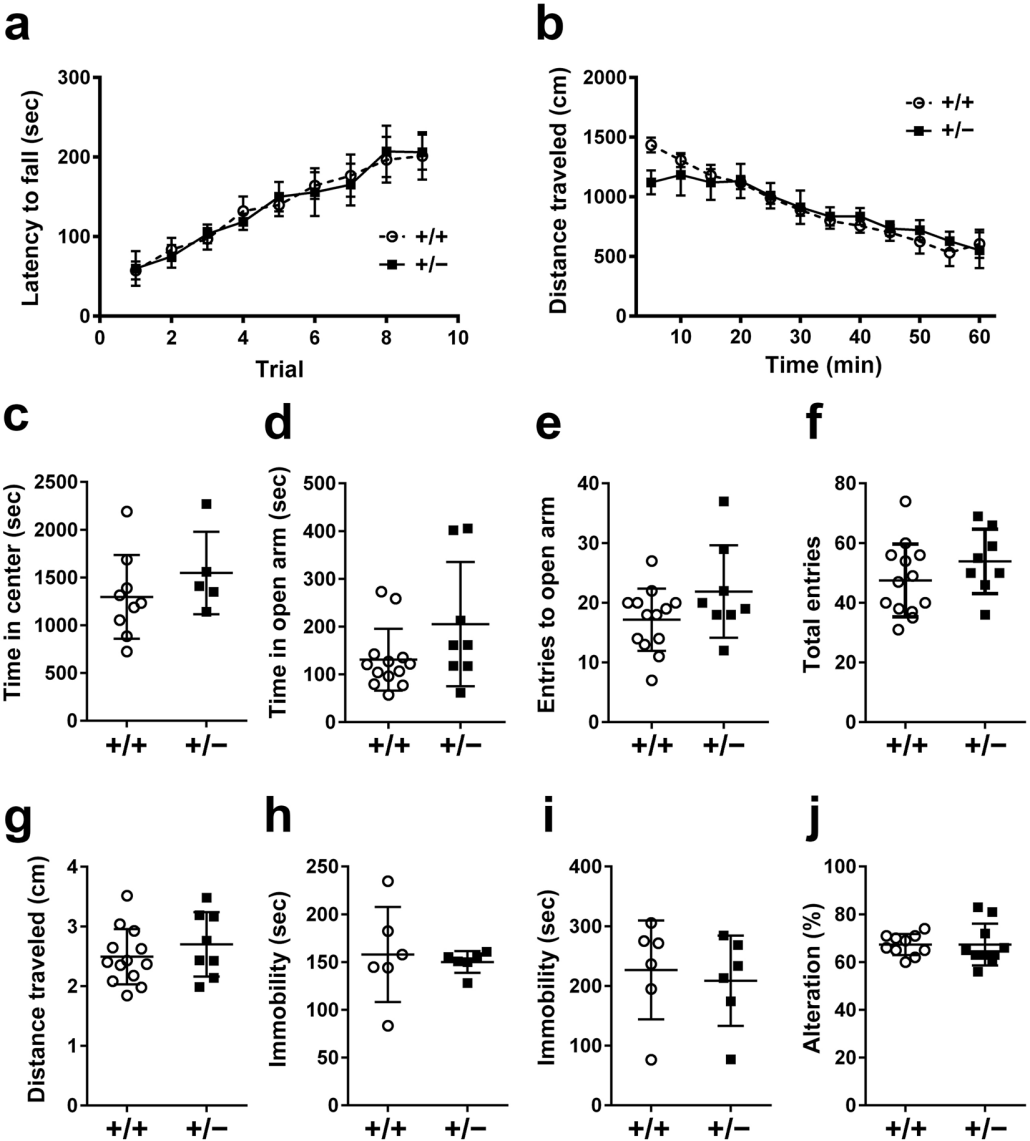
A comprehensive behavioral test battery for *miR-124-1*^+/−^ mice. (**a**) Rotarod test of *miR-124-1*^+/−^ mice for analysis of motor function integrity (WT, n = 9; Het n = 5). Latency to fall was measured. (**b**,**c**) In open field, locomotor activity and anxiety-like behavior of WT control and *miR-124-1*^+/−^ mice were measured based on the distance traveled and time in center (WT, n = 9; Het, n = 5). (**d**–**g**) Elevated plus maze test of *miR-124-1*^+/−^ mice for analyzing the anxiety-like behavior. A cumulative time spent in the open arm, a number of entries to the open arm, total entries, and total distance moved were monitored (WT, n = 13; Het, n = 8). (**h**) Tail suspension test of *miR-124-1*^+/−^ mice for analysis of depression-like behavior. Immobility time was measured (n = 6 per genotype). (**i**) Forced swim test of *miR-124-1*^+/−^ mice for analysis of depression-like behavior. Immobility time was measured (n = 6 per genotype). (**j**) Y-maze test of *miR-124-1*^+/−^ mice for a spatial working memory task based on the natural tendency of mice to alternate the selection of maze arms. Percentages of spontaneous alternation was measured (n = 10 per genotype). Error bars represent ± SD.

To address whether *miR-124-1* haploinsufficiency affects normal brain functions, we subjected *miR-124-1*^+/−^ mice to a comprehensive behavioral test battery. In the rotarod test, *miR-124-1*^+/−^ mice exhibited normal motor function (Fig. 1a; two-way repeated-measures ANOVA: Genotype: *F*_(1, 12)_ = 0.001206, *p* = 0.9729; Time: *F*_(8, 96)_ = 19.94, *p* < 0.0001; Interaction: *F*_(8, 96)_ = 0.1703, *p* = 0.9943). We monitored locomotor activity of the *miR-124-1*^+/−^ mice in the open field test, and observed no significant difference in total distance traveled compared with WT control mice (Fig. 1b; two-way repeated-measures ANOVA: Genotype: *F*_(1, 12)_ = 0.02964, *p* = 0.8662; Time: *F*_(11, 132)_ = 18.44, *p* < 0.0001; Interaction: *F*_(11, 132)_ = 0.9734, *p* = 0.4738). To assess anxiety-like behavior, we monitored time spent in the central part of the open field. We also measured the number of entries to the open arm and cumulative time spent in the open arm of the elevated plus maze. There were no significant differences in these parameters between *miR-124-1*^+/−^ and WT mice (Fig. 1c–g; unpaired *t* test: t_(12)_ = 1.029, *p* = 0.3236; unpaired *t* test: t_(19)_ = 1.752, *p* = 0.0959; unpaired *t* test: t_(19)_ = 1.676, *p* = 0.1101; unpaired *t* test: t_(19)_ = 1.219, *p* = 0.2377; unpaired *t* test: t_(19)_ = 0.9325, *p* = 0.3628). To test whether *miR-124-1* haploinsufficiency contributes to depression-like behavior, we subjected *miR-124-1*^+/−^ mice to tail suspension and forced swim tests. *miR-124-1*^+/−^ mice exhibited no significant difference in immobility time (Fig. 1h,i; unpaired *t* test: t_(10)_ = 0.3745, *p* = 0.7518; unpaired *t* test: t_(10)_ = 0.3952, *p* = 0.7010). To test for short-term memory deficit, we conducted a test of spontaneous Y-maze alternations, a spatial working memory task based on the natural tendency of mice to alternate the selection of maze arms. We did not observe differences in spontaneous alternation between *miR-124-1*^+/−^ and WT mice (Fig. 1j; unpaired *t* test: t_(18)_ = 0.00, *p* > 0.9999). These results suggest that *miR-124-1* haploinsufficiency does not affect normal motor function, anxiety-like behavior, depression-like behavior, or spatial working memory. The previous study shows that *miR-124-1*^−/−^ mice exhibit a significantly decreased number of cone photoreceptor cells and an impairment of photopic electroretinogram (ERG) responses^2^. We measured the ERG responses from *miR-124-1*^+/−^ mice and observed no substantial ERG abnormalities (Supplementary Material Fig. S1a–c). We also analyzed cone photoreceptor integrity in the *miR-124-1*^+/−^ retina by immunofluorescent staining and observed no significant differences between *miR-124-1*^+/−^ and control retinas (Supplementary Material Fig. S1d). These results suggest that *miR-124-1*^+/−^ mice have no substantial defects in the retina.

### Haploinsufficiency of *miR-124-1* causes abnormalities in social, psychostimulant induced behaviors, and sensorimotor gating

**Figure 2:**
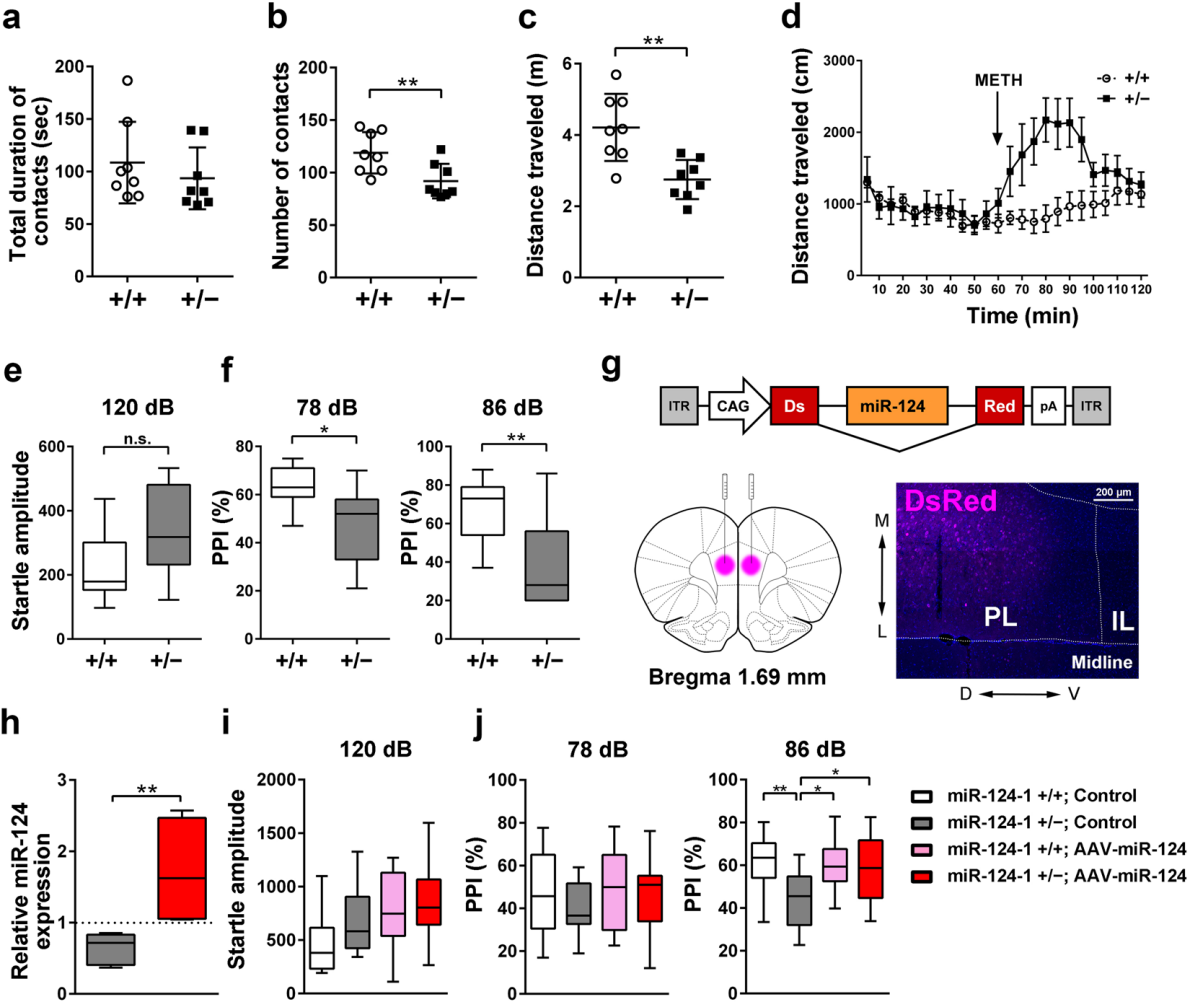
*miR-124-1* haploinsufficiency results in behavioral deficits. (**a**–**c**) Sociability of *miR-124-1*^+/−^ mice was measured based on interaction duration (**a**), the number of contacts (**b**) (n = 8 per genotype; ^**^p < 0.01), and total distance traveled during social interaction (**c**) (n = 8 per genotype; ^**^p < 0.01). (**d**) Methamphetamine induced hyperactivity in *miR-124-1*^+/−^ mice but not in WT control mice (WT, n = 10; Het, n = 5). (**e**,**f**) PPI in *miR-124-1*^+/−^ mice was impaired, whereas startle response amplitude was not significantly changed compared with that in WT mice (WT, n = 11; Het, n = 7; ^*^p < 0.05, ^**^p < 0.01; n.s., not significant). (**g**) A schematic of the AAV construct expressing *miR-124* and *Ds-Red* (AAV-DsRed-miR-124). AAV-DsRed-miR-124 was injected into the PFC of 2 M WT and *miR-124-1*^+/−^ mice. Fluorescence indicates the brain section injected with AAV-DsRed-miR-124. L, lateral; M, medial; D, dorsal; V, ventral; PL, prelimbic; IL, infralimbic. (**h**) Quantification of *miR-124* expression level in the PFC of AAV-DsRed-miR-124-injected *miR-124-1*^+/−^ mice (control AAV-DsRed injected, n = 7; AAV-DsRed-miR-124 injected, n = 4; ^**^p < 0.01). The dotted line indicates *miR-124* expression level in control AAV-DsRed-injected WT mice. (**i**,**j**) Injection of AAV-DsRed-miR-124 into the PFC rescued the PPI deficit in *miR-124-1*^+/−^ mice (n = 12-15 per group; ^*^p < 0.05, ^**^p < 0.01). Error bars in (**a**–**d**) represent ± SD. Box–whisker plots present median (center line), ±1.5 interquartile range (box), minimal and maximal values (whiskers) in (**e**,**f**,**h**–**j**).

Since social deficits often appear in psychiatric disorders^20^, we examined the sociability of *miR-124-1*^+/−^ mice by social interaction test. Although there was no significant difference in the total duration of contacts between *miR-124-1*^+/−^ and WT control mice, the total number of contacts for *miR-124-1*^+/−^ mice decreased significantly compared to that of WT mice (Fig. 2a,b; unpaired *t* test: t_(14)_ = 0.8695, *p* = 0.3993; unpaired *t* test: t_(14)_ = 2.980, *p* = 0.0099). In addition, although the spontaneous locomotor activity in *miR-124-1*^+/−^ mice was unchanged in the open field test (Fig. 1b), the total distance traveled in social interaction was significantly decreased (Fig. 2c; unpaired *t* test: t_(14)_ = 3.791, *p* = 0.0020). These results suggest that *miR-124-1* haploinsufficiency causes social behavior deficits.

A dopaminergic psychostimulant, methamphetamine, raises dopamine levels in the nucleus accumbens and frontal cortex, and increases locomotor activity^21^. To examine whether *miR-124-1*^+/−^ mice show dopamine-associated behavioral deficits, we investigated the response of *miR-124-1*^+/−^ mice to methamphetamine using the open field. We found that the distance traveled by *miR-124-1*^+/−^ mice in the open field test increased significantly compared to WT mice after methamphetamine administration (Fig. 2d; two-way repeated-measures ANOVA: Genotype: *F*_(1, 13)_ = 6.714, *p* = 0.0224; Time: *F*_(12, 156)_ = 4.304, *p* < 0.0001; Interaction: *F*_(12, 156)_ = 6.334, *p* < 0.0001). This result suggests that *miR-124-1* haploinsufficiency affects dopamine signaling associated with psychostimulant-induced hyperactivity.

Prepulse inhibition (PPI) is a reduction in an acoustic startle response observed when a weak stimulus (prepulse) is presented before the startling stimulus^22^. PPI is a measure of sensorimotor gating that is impaired in various neuropsychiatric disorders, including schizophrenia, Huntington’s disease, Tourette’s disease^23^, autism^24^, and panic disorder^25^. To test whether *miR-124-1* haploinsufficiency affects sensorimotor gating, we measured PPI in *miR-124-1*^+/−^ mice. We observed that the amplitudes of acoustic startle response were similar in *miR-124-1*^+/−^ and WT mice (Fig. 2e; unpaired *t* test: t_(16)_ = 2.046, *p* = 0.0576). In contrast, we found that the percentages of PPI both with 78 dB and 86 dB prepulses decreased significantly in *miR-124-1*^+/−^ mice (Fig. 2f; unpaired *t* test: t_(16)_ = 2.566, *p* = 0.0207; unpaired *t* test: t_(16)_ = 2.745, *p* = 0.0144), suggesting that *miR-124-1* haploinsufficiency results in a deficit of sensorimotor gating in mice.

### Partial rescue of the PPI deficit in *miR-124-1*^+/−^ mice

We observed multiple types of behavior deficits in *miR-124-1*^+/−^ mice: social deficits, methamphetamine-induced hyperactivity, and impairment of PPI. Since all of these phenotypes are associated with the impairment of prefrontal cortex (PFC) functioning^26–28^, we focused on the role of *miR-124-1* in the PFC. First, we examined expression of *miR-124* in the PFC from *miR-124-1*^+/−^ and WT control mice at 1-month old (M) and 2 M by qRT-PCR analysis, and found that the *miR-124* levels significantly decreased in the *miR-124-1*^+/−^ PFC compared with those in the WT PFC at both stages (Supplementary Material Fig. S1e,f). To determine whether downregulation of *miR-124* contributes to the impairment of PFC function in *miR-124-1*^+/−^ mice, we tested whether the impaired PPI in *miR-124-1*^+/−^ mice can be rescued by overexpression of *miR-124* in the PFC. We prepared an adeno-associated virus (AAV) expressing DsRed and *miR-124* (AAV-DsRed-miR-124) (Fig. 2g). We injected AAV-DsRed-miR-124 or control AAV-DsRed into the PFC of 2 M WT and *miR-124-1*^+/−^ mice, and measured PPI at 2 weeks (wks) after AAV injection. We confirmed that injection of AAV-DsRed-miR-124 significantly increases the expression level of *miR-124* in the PFC of *miR-124-1*^+/−^ mice (Fig. 2h; Mann–Whitney U test: *p* = 0.0061). Injection of AAV-DsRed-miR-124 or control AAV-DsRed into the PFC of WT mice did not affect the amplitudes of the acoustic startle response or PPI (Fig. 2i; two-way ANOVA: Genotype: *F*_(1, 49)_ = 2.301, *p* = 0.1357; Virus: *F*_(1, 49)_ = 7.740, *p* = 0.0076; Interaction: *F*_(1, 49)_ = 0.7856, *p* = 0.3798). In contrast, the impaired PPI with an 86 dB prepulse in *miR-124-1*^+/−^ mice recovered with the injection of AAV-DsRed-miR-124 (Fig. 2j; two-way ANOVA: Genotype: *F*_(1, 49)_ = 1.161, *p* = 0.2865; Virus: *F*_(1, 49)_ = 0.4301, *p* = 0.5150; Interaction: *F*_(1, 49)_ = 0.2737, *p* = 0.6032; two-way ANOVA: Genotype: *F*_(1, 49)_ = 7.184, *p* = 0.0100; Virus: *F*_(1, 49)_ = 2.768, *p* = 0.1025; Interaction: *F*_(1, 49)_ = 5.393, *p* = 0.0244). These results suggest that the expression level of *miR-124* in the PFC is critical for normal sensorimotor gating in mice.

### *miR-124-1* haploinsufficiency enhances synaptic transmission in layer 5 pyramidal cells in the PFC

**Figure 3:**
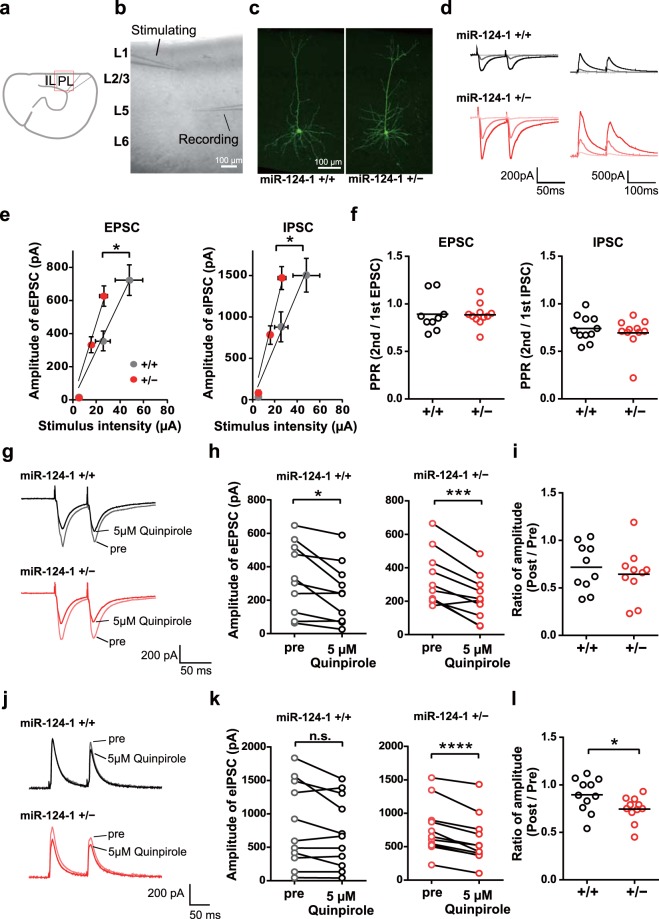
The effect of *miR-124-1* haploinsufficiency on synaptic transmissions in layer 5 pyramidal cells in the PFC. (**a**) A schematic representing the recording region (red square) in PFC slices. PL, prelimbic, IL, infralimbic. (**b**) IR-DIC image of the PL region and the location of stimulating and recording electrodes. (**c**) Representative images showing biocytin-filled layer 5 pyramidal cells in WT control (left) and *miR-124-1*^+/−^ mice (right). (**d**) Example average (n = 20) traces of EPSCs (left) and IPSCs (right) evoked by paired-pulse electrical stimulation at three stimulus intensities in WT (upper, black) and *miR-124-1*^+/−^ mice (lower, red). (**e**) The average peak amplitude of evoked EPSCs (left) and IPSCs (right) was plotted against the stimulus intensity. Vertical and horizontal error bars indicate the SEM of the amplitude of evoked responses and the intensity of stimulation, respectively (n = 11 per genotype; ^*^p < 0.05). (**f**) Paired-pulse ratio (PPR) of EPSCs (left) and IPSCs (right), respectively (WT, n = 9; Het = 11). (**g**) Example average (n = 20) traces of EPSCs before (pre, lighter color) and during (darker color) quinpirole application in WT (upper, black) and *miR-124-1*^+/−^ (lower, red) mice. (**h**) Plots of the peak amplitude of EPSCs before (pre) and during quinpirole application in WT (black, left) and *miR-124-1*^+/−^ (red, right) mice (n = 10 per genotype; ^*^p < 0.05, ^***^p < 0.001). (**i**) The ratio of EPSC amplitude before and during quinpirole application in WT (black, left) and *miR-124-1*^+/−^ (red, right) mice. (**j**) Example average (n = 20) traces of IPSCs before (pre, lighter color) and during (darker color) quinpirole application in WT (upper, black) and *miR-124-1*^+/−^ (lower, red) mice. (**k**) Plots of the peak amplitude of IPSCs before (pre) and during quinpirole application in WT (black, left) and *miR-124-1*^+/−^ (red, right) mice (n = 11 per genotype; ^****^p < 0.0001; n.s., not significant). (**l**) The ratio of IPSC amplitude before and during quinpirole application in WT (black, left) and *miR-124-1*^+/−^ (red, right) mice (^*^p < 0.05).

To investigate the effect of *miR-124-1* haploinsufficiency on synaptic transmission in the PFC, we conducted whole-cell recordings from layer 5 pyramidal neurons in PFC slices prepared from *miR-124-1*^+/−^ mice (Fig. 3a–c). To make a comparison of the strength of excitatory and inhibitory synaptic inputs to the pyramidal cells between the WT control and *miR-124-1*^+/−^ mice, we recorded excitatory postsynaptic currents (EPSCs) at −70 mV and inhibitory postsynaptic currents (IPSCs) at 0 mV in response to electrical stimulation of presynaptic fibers at three different intensities (Fig. 3d). The slopes of the stimulus-response curves for EPSCs and IPSCs were both significantly steeper in *miR-124-1*^+/−^, compared with WT mice (Fig. 3e; unpaired *t* test: t_(19)_ = 2.301, *p* = 0.0329; unpaired *t* test: t_(20)_ = 2.611, *p* = 0.0167). There were no significant differences in the paired-pulse ratio of EPSCs or IPSCs between WT and *miR-124-1*^+/−^ mice (Fig. 3f; unpaired *t* test: t_(18)_ = 0.08295, *p* = 0.9348; unpaired *t* test: t_(20)_ = 0.7042, *p* = 0.4894). These results suggest that both excitatory and inhibitory synaptic transmissions in layer 5 pyramidal neurons are enhanced by *miR-124-1* heterozygous deficiency.

We observed hypersensitivity of locomotor activity induced by methamphetamine administration and impaired PPI in *miR-124-1*^+/−^ mice (Fig. 2d,f). Previous studies have reported that absence of *Dopamine D2 receptor (Drd2)* attenuates the disruption of PPI induced by amphetamine^29^, and methamphetamine-induced hyperactivity^30^. To investigate whether dopamine regulation of local circuit processing in the PFC is affected in *miR-124-1*^+/−^ mice, we examined the effects of Drd2 activation on synaptic transmission in *miR-124-1*^+/−^ mice. We observed a significant reduction in EPSC amplitude after the application of 5 μM quinpirole, a Drd2 agonist, in both *miR-124-1*^+/−^ mice and WT mice, but no significant difference in the level of reduction between them (Fig. 3g–i; paired *t* test: t_(9)_ = 3.165, *p* = 0.0115; paired *t* test: t_(9)_ = 5.436, *p* = 0.0004; unpaired *t* test: t_(18)_ = 0.6279, *p* = 0.5379). On the other hand, the amplitude of IPSCs significantly decreased by quinpirole application in *miR-124-1*^+/−^ mice but not in WT mice (Fig. 3j–l; paired *t* test: t_(10)_ = 2.054, *p* = 0.0670; paired *t* test: t_(10)_ = 6.770, *p* < 0.0001; unpaired *t* test: t_(20)_ = 2.213, *p* = 0.0387). These results suggest that Drd2-mediated dopaminergic modulatory effects on inhibitory transmissions increased in the pyramidal cells in the PFC of *miR-124-1*^+/−^ mice. We speculated that PPI deficit and methamphetamine-induced hyperactivity observed in *miR-124-1*^+/−^ mice are likely due to modulation of the dopaminergic system by *miR-124* reduction in the PFC.

### *Drd2* is up-regulated in the *miR-124-1*^+/−^ PFC

**Figure 4:**
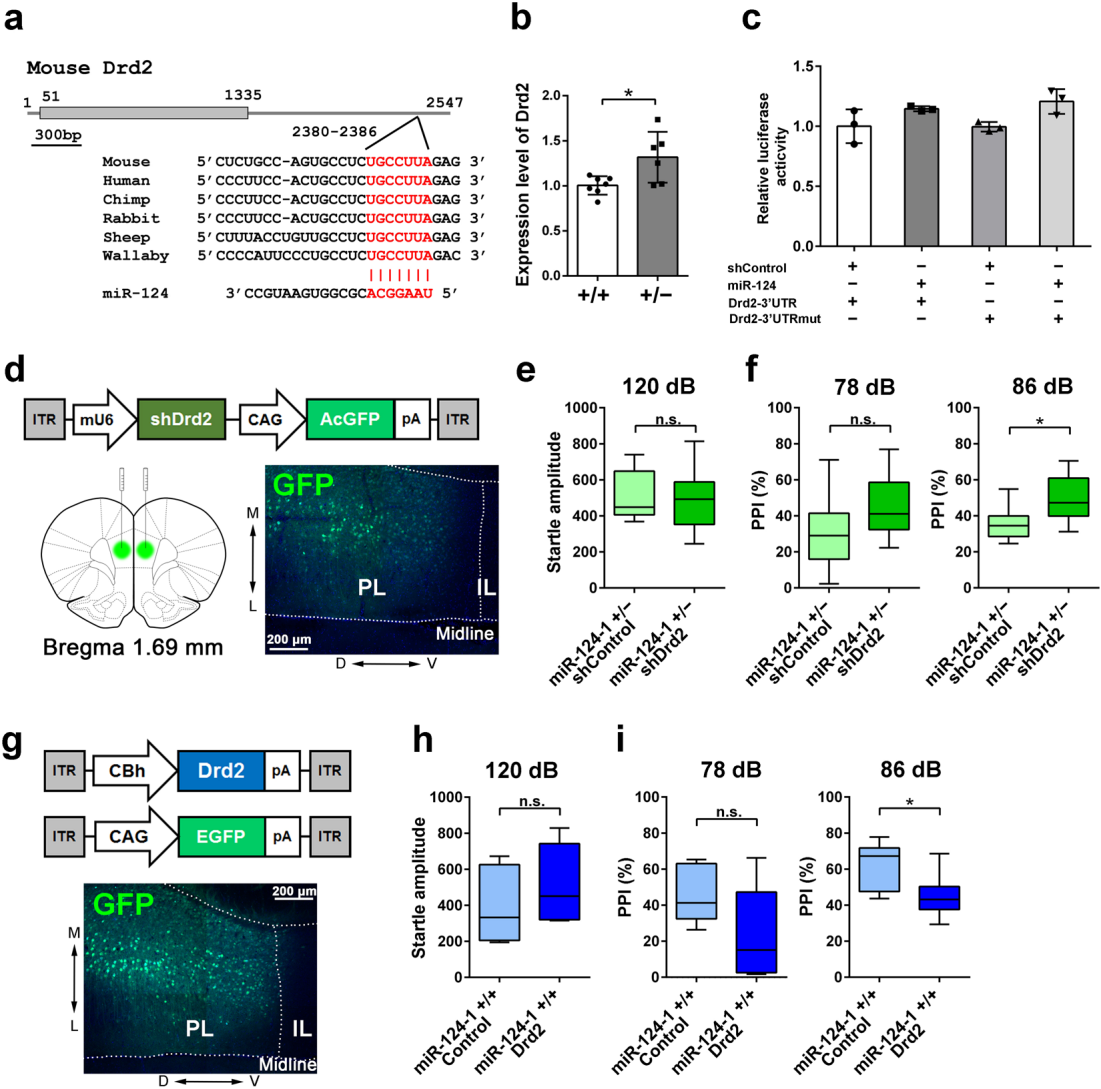
Effect of *Drd2* expression level in the PFC to PPI deficit. (**a**) Drd2 3′UTR contains a *miR-124* target site. A *miR-124* target site in *Drd2* 3′UTR was predicted by TragetScan. This target site is conserved among several mammalian species. (**b**) *Drd2* expression level in the *miR-124-1*^+/−^ PFC is quantified by qRT-PCR (WT, n = 7; Het, n = 6; ^*^p < 0.05). (**c**) Luciferase reporter assay of *Drd2*-3′UTR containing a *miR-124* target sequence. A *miR-124* expression plasmid was transfected into HEK293 cells with reporter plasmids containing Drd2-3′UTR or Drd2-3′UTRmut (n = 3 per group). After transfection, the cells were incubated for 48 h and the luminescence signal was measured. (**d**) A schematic of AAV construct expressing shRNA for *Drd2* and *AcGFP* (AAV-shDrd2). AAV-shDrd2 was injected into the 2 M *miR-124-1*^+/−^ PFC. GFP-positive cells were observed in the PFC. L, lateral; M, medial; D, dorsal; V, ventral. PL, prelimbic, IL, infralimbic. (**e**,**f**) *Drd2* knockdown partially rescued PPI deficit in the *miR-124-1*^+/−^ mice. Startle response amplitude (**e**) and PPI (**f**) of AAV-shDrd2-injected mice were analyzed (shControl, n = 8; shDrd2, n = 9; ^*^p < 0.05; n.s., not significant). (**g**) A schematic of AAV construct expressing *Drd2* (AAV-Drd2) or *EGFP* (AAV-GFP). AAV-Drd2 and -GFP were coinjected into the WT PFC. (**h**,**i**) Drd2 overexpression in the PFC causes PPI deficit similar to that observed in *miR-124-1*^+/−^ mice. Startle response amplitude (**h**) and PPI (**i**) of AAV-Drd2-injected mice were analyzed. (Control, n = 6; Drd2, n = 7; ^*^p < 0.05; n.s., not significant). Error bars in (**b**,**c**) represent ± SD. Box–whisker plots present median (center line), ±1.5 interquartile range (box) and minimal and maximal values (whiskers) in (**e**,**f**,**h**,**i**).

To explore the molecular mechanism of behavioral deficits observed in *miR-124-1*^+/−^ mice, we focused on genes involved in the dopaminergic system. We searched for *miR-124* target sites in 3′UTR of dopamine-related genes, including dopamine receptor genes (*Drd1-5*), a dopamine transporter gene (*DAT*), a dopamine synthesis gene (*TH*), and a dopamine metabolism gene (*MAOB*), and found that only the 3′UTR of *Drd2* possesses an evolutionally conserved *miR-124* target sequence (Fig. 4a). We observed that total *Drd2* (containing both long and short variants) increased in 2 M *miR-124-1*^+/−^ PFC (Fig. 4b; unpaired *t* test: t_(11)_ = 2.749, *p* = 0.0189).

To examine the targeting effect of *miR-124* on the *Drd2*-3′UTR, we performed a luciferase reporter assay. We prepared reporter plasmids containing native *Drd2*-3′UTR (Drd2-3′UTR) and *Drd2*-3′UTR with mutations in the *miR-124* target site (Drd2-3′UTRmut) (Fig. 4c). We observed that luciferase activity shows no obvious change between the cells transfected with Drd2-3′UTR and those transfected with Drd2-3′UTRmut (one-way ANOVA: *F*_(3, 8)_ = 4.156, *p* = 0.0476). This result suggests that *Drd2* is not a direct target of *miR-124*, but is indirectly regulated by *miR-124* in the PFC.

### Expression level of *Drd2* in the PFC affects PPI

To test whether *Drd2* suppression in the *miR-124-1*^+/−^ PFC rescues the PPI, we used AAVs expressing *shDrd2* to knockdown *Drd2* (AAV-shDrd2) or a control shRNA (AAV-shControl)^31^ (Fig. 4d). We injected AAV-shDrd2 into the *miR-124-1*^+/−^ PFC at 2 M and measured PPI at 2 wks after injection. Acoustic startle amplitude was unchanged between AAV-shControl- or AAV-shDrd2-injected mice (Fig. 4e; Mann–Whitney U test: *p* = 0.9336). No significant change of PPI with a 78 dB prepulse was observed in these mice, in contrast, a significant recovery of the PPI deficit was observed by knockdown of *Drd2* in the PFC with an 86 dB prepulse (Fig. 4f; Mann–Whitney U test: *p* = 0.1650; Mann–Whitney U test: *p* = 0.0274). These results suggest that the impaired PPI in *miR-124-1*^+/−^ mice is at least partially caused by *Drd2* increase in the PFC.

To investigate whether *Drd2* overexpression in the PFC affects PPI, we prepared AAVs expressing *Drd2* (AAV-Drd2) or EGFP (AAV-GFP), and co-injected them into the PFC of WT mice at 2 M (Fig. 4g). We observed no significant change of startle amplitude between control AAV-GFP- and AAV-Drd2-injected mice (Fig. 4h; Mann–Whitney U test: *p* = 0.2890). No significant change of PPI with a 78 dB prepulse was observed in these mice, however, we observed a significant PPI decrease with an 86 dB prepulse in mice with *Drd2* overexpression by AAV-Drd2 injection into the PFC (Fig. 4i; Mann–Whitney U test: *p* = 0.1375; Mann–Whitney U test: *p* = 0.0350). These results suggest that *Drd2* level change is at least partially responsible for the sensorimotor gating abnormality in the PFC of *miR-124-1*^+/−^ mice.

**Figure 5:**
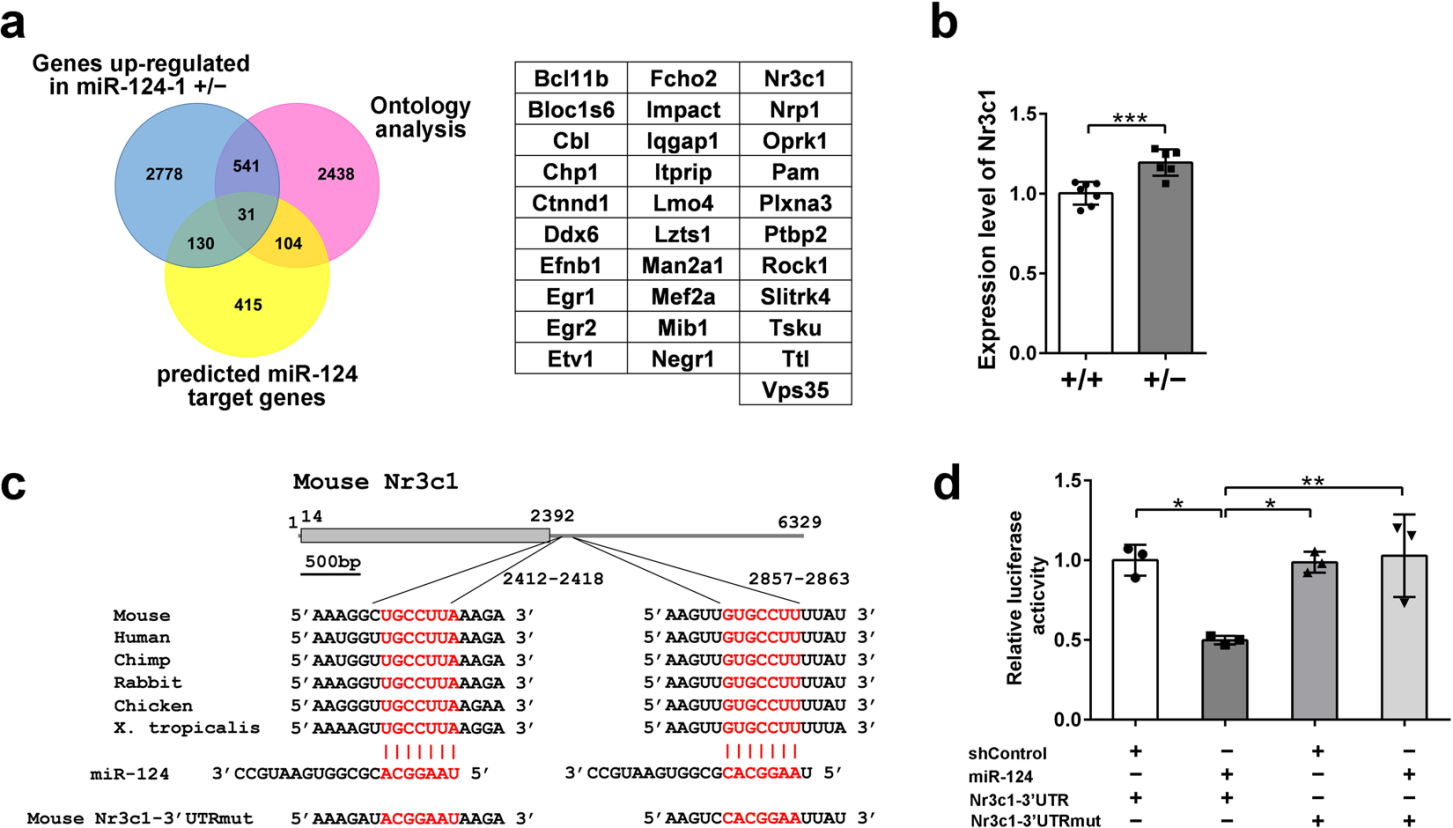
Identification of *Nr3c1* as an *in vivo* target gene of *miR-124*. (**a**) Venn diagram indicating up-regulated genes in the *miR-124-1*^+/−^ PFC (blue circle), predicted *miR-124* target genes (yellow circle), and neuron-associated genes classified by ontology analysis (pink circle). *miR-124* target genes were identified by TragetScan (cumulative weighted context++ score > −0.25). A list of the 31 genes at the intersection of the Venn diagram. (**b**) *Nr3c1* expression increased in the *miR-124-1*^+/−^ PFC. The *Nr3c1* expression level was measured by qRT-PCR (WT, n = 7; Het, n = 6; ^***^p < 0.001). (**c**) A schematic showing a predicted *miR-124* target gene *Nr3c1*. Predicted target sites are conserved in vertebrates. Two *miR-124* target sequences are shown in red. (**d**) Luciferase reporter assays of *Nr3c1*-3′UTR. HEK293 cells were transfected with reporter plasmids of Nr3c1-3′UTR or Nr3c1-3′UTRmut with the *miR-124* expression plasmid. (n = 3 per group; ^*^p < 0.05, ^**^p < 0.01). Error bars in (**b**,**d**) represent ± SD.

In the reporter assay, we did not observe a significant change in luciferase activity between native and mutated *Drd2*-3′UTR constructs (Fig. 4c). How was *Drd2* expression enhanced in the *miR-124-1*^+/−^ PFC? To address this question, we further screened *miR-124* target genes in the PFC. To identify *miR-124* target genes in the PFC, we performed high-throughput mRNA sequencing (RNA-seq) analysis using RNAs isolated from *miR-124-1*^+/−^ PFC or control PFC at 2 M (Fig. 5a). We identified 3,480 upregulated genes (more than 1.1-fold increase) in the *miR-124-1*^+/−^ PFC. We next searched for putative target genes of *miR-124* in the context of PFC development. We identified 682 genes containing predicted *miR-124* binding sites in their 3′UTR using TargetScan (http://www.targetscan.org)^32^. We identified 3,114 genes with expected function in neurons using gene ontology terms. We identified 31 genes that overlap in these three criteria (Fig. 5a). We selected several genes from these candidates and confirmed increased expression of them in the *miR-124-1*^+/−^ PFC by qRT-PCR. Among them, we focused on *Nr3c1* (a glucocorticoid receptor), which enhances *Drd2* expression in the frontal cortex of *Disc1* transgenic mice^33^. By qRT-PCR we confirmed that *Nr3c1* expression is significantly up-regulated in the *miR-124-1*^+/−^ PFC (Fig. 5b; unpaired *t* test: t_(11)_ = 4.509, *p* = 0.0009). *Nr3c1*-3′UTR has two *miR-124* target sites that are highly conserved in vertebrates (Fig. 5c). To examine whether *Nr3c1* is directly regulated by *miR-124*, we carried out a luciferase assay using the *Nr3c1*-3′UTR constructs (Fig. 5d). Luciferase activity with the native *Nr3c1*-3′UTR sequence was significantly down-regulated by cotransfection with the *miR-124* expression plasmid when compared to that with mutated *Nr3c1*-3′UTR (Nr3c1-3′UTRmut) (one-way ANOVA: *F*_(3,8)_ = 0.7195, *p* = 0.0051). These results suggest that *Nr3c1* is directly regulated by *miR-124* in the PFC. *Drd2* is likely to be regulated indirectly in the PFC by *miR-124* through regulation of *Nr3c1* expression change.

Several studies reported that *miR-124* plays roles in pathogenesis of psychiatric disorders including Alzheimer’s disease and frontotemporal dementia (FTD)^28,34^. In their studies, human brains with Alzheimer’s disease exhibited a slight decrease of *miR-124* expression, and the FTD mouse model with an impairment of age-dependent sociability also exhibited *miR-124* down-regulation in the PFC. The impaired sociability of the FTD mouse model was shown to be accompanied with up-regulation of AMPA receptor subunits (*Gria2, 3* and 4) following *miR-124* down-regulation. We examined expression of *Gria2*, 3 and 4 in the 2 M *miR-124-1*^+/−^ PFC by qRT-PCR, however, the expression of these genes was not significantly altered (Supplementary Material Fig. S2a; unpaired *t* test: t_(11)_ = 1.461, *p* = 0.1720; unpaired *t* test: t_(11)_ = 0.9990, *p* = 0.3393; unpaired *t* test: t_(11)_ = 0.5248, *p* = 0.6101). A recent study reported that mice exposed to chronic ultra-mild stress exhibited depression-like behaviors and decreased *miR-124* expression in the hippocampus (approximately 80% of non-stressed control mice)^35^. The reduction of *miR-124* expression in the hippocampus caused by chronic ultra-mild stress induced the up-regulation of *Glycogen synthase kinase 3β* (*Gsk3b*). We measured expression of *miR-124* and *Gsk3b* in the 2 M *miR-124-1*^+/−^ hippocampus by qRT-PCR. We observed a decrease of *miR-124* (approximately 73% of WT control mice, Supplementary Material Fig. S2b; unpaired *t* test: t_(4)_ = 3.805, *p* = 0.0190) but no significant change of *Gsk3b* in the *miR-124-1*^+/−^ hippocampus was detected (Supplementary Material Fig. S2c; unpaired *t* test: t_(4)_ = 0.8932, *p* = 0.4222). Another study showed that small GTPase regulatory gene *EPAC*-deficient mice, which exhibit an impairment of learning and social interaction, displayed an elevation of *miR-124* expression in the cortex and hippocampus, leading to down-regulation of *miR-124* target gene *Zif268* (*Egr1*)^36^. We did not observe a significant expression change of *Zif268* in the 2 M *miR-124-1*^+/−^ PFC and hippocampus by qRT-PCR analysis (Supplementary Material Fig. S2d; unpaired *t* test: t_(11)_ = 1.631, *p* = 0.1312; unpaired *t* test: t_(4)_ = 0.5992, *p* = 0.5813). Thus, the expression levels of the previously reported *miR-124* target genes, *Gria2, Gria3, Gria4, Gsk3b*, and *Zif268*, in the *miR-124-1*^+/−^ PFC were not significantly affected compared to those in the control PFC. Since the experimental design of these studies is different from our study^28,35,36^, future studies are needed to elucidate the cause of varied outcomes.

## Discussion

In the current study, several lines of evidence suggest that impaired sensorimotor gating in *miR-124-1*^+/−^ mice is likely due to abnormal dopaminergic system by *miR-124* reduction in the PFC. First, our whole-cell recordings from layer 5 pyramidal cells in the *miR-124-1*^+/−^ PFC revealed that the amplitude of IPSCs significantly decreased by Drd2 agonist quinpirole application. Second, we found that *Drd2* expression is upregulated in the *miR-124-1*^+/−^ PFC. Third, injection of AAV-shDrd2 into the PFC of *miR-124-1*^+/−^ mice partially rescued PPI impairment. Fourth, overexpression of *Drd2* in the WT PFC by injection of AAV-Drd2 caused a PPI abnormality. In addition, a previous study reported that injection of dopamine receptor agonist apomorphine into the mouse PFC significantly compromises PPI^37^. Accordingly, our results suggest the aberrant Drd2 pathway in the PFC is a major cause of sensorimotor gating deficit observed in *miR-124-1*^+/−^ mice.

Several previous studies reported *in vivo* microRNA functions in normal brain using mouse mutants. In the striatum, *miR-128* regulates motor behavior by modulating the ERK signaling network and neuronal excitability^38^. *miR-137*, a schizophrenia risk gene product, controls synaptic plasticity and memory in the hippocampus^39^. *miR-338-3p* in the auditory thalamus regulates auditory thalamus development and acoustic-startle response^40^. In the current study, we showed that *miR-124* is necessary for the normal brain function in the PFC. Whole-cell recordings from layer 5 pyramidal neurons in *miR-124-1*^+/−^ PFC slices revealed that synaptic transmission into pyramidal neurons in the PFC is enhanced by *miR-124-1* heterozygous deficiency. Previous studies showed the link between function of pyramidal neurons in the PFC and animal behavior. Loss of *Dysbindin-1*, whose genetic variation is associated with risk for schizophrenia, shows impairments in working memory, PPI, and higher locomotor activity. In the *Dysbindin-1* mutant PFC, enhanced excitability of pyramidal neurons was observed^41^. *DISC1* knockdown in the mouse PFC leads to deficits in PPI and methamphetamine-induced hyperactivity^27^. In *DISC1* knockdown mice, attenuated electrophysiological responsiveness of pyramidal neurons in the PFC was observed. The mice lacking GluN2C, an NMDA receptor subunit, exhibited abnormalities in cognition and PPI as well as activity alteration of layer 5 pyramidal neurons in the PFC^42^. Since none of *Dysbindin-1, DISC1* and *GluN2C* transcripts contain the *miR-124* target sequence, we speculate that these genes function in distinct pathways from that of *miR-124* in normal brain function. We cannot rule out the possibility that the abnormal electrophysiological properties of pyramidal neurons in the *miR-124-1*^+/−^ PFC are due to indirect effects from other cells. Future analysis using PFC pyramidal neuron specific-*miR-124* conditional knock-out mice may clarify this point.

It was previously shown that Nr3c1 3′ UTR contains a *miR-124* target sequence and that luciferase reporter expression was downregulated by *miR-124* in a cultured cell system^43^. In the current study, we observed upregulation of *Nr3c1* by reduction of *miR-124 in vivo*, suggesting that *Nr3c1* is an endogenous target of *miR-124* in the prefrontal cortex. Blocking of *Nr3c1* function decreases the Drd2 expression level, and affects the dopaminergic pathway in the frontal cortex of one mouse model of adolescent stress^33^. Conditional deletion of *Nr3c1* in dopaminoceptive neurons affects the motivation of mice to self-administer cocaine^44^. These observations as well as on our luciferase reporter assay suggest that *Drd2* is regulated indirectly in the PFC by *miR-124* through modulation of *Nr3c1* expression change. To examine this point *in vivo*, we performed injection of an Nr3c1 antagonist into the *miR-124-1*^+/−^ PFC, however, we did not observe a statically significant change of PPI or an expression change of the *Drd2* mRNA by RT-qPCR analysis using the whole PFC. It is possible that the *Drd2* expression in the PFC is redundantly regulated by Nr3c1 and other molecules. There might be another possibility that only a subset of PFC neuronal cells is regulated by Nr3c1 and the expression change of *Drd2* might be difficult to be detected in the whole PFC by RT-qPCR analysis.

Heterozygous deletions on human 8p23.1 containing *miR-124-1* locus are associated with psychiatric disorders^15–18^. Interestingly, a recent genome-wide association study reported that the inversion polymorphism in the 8p23.1 region containing the human *miR-124-1* locus is associated with neuroticism^45^. Further investigation of *miR-124* mutations and/or expression levels in human neuropsychiatric disorder patients, including schizophrenia, Alzheimer’s disease, FTD, autism, and social impairment, may advance our understanding on the pathogenesis and/or exacerbation of these diseases.

## Materials and Methods

### Animal care

All recombinant mouse experiment procedures were approved by the Institutional Safety Committee on Recombinant DNA Experiments (approval ID 04220), Animal Experimental Committees of the Institute for Protein Research at Osaka University (approval ID 29-01-2), Committee on Animal Research at Kyoto University Graduate School of Medicine (approval ID MedKyo17071), and Experimental Animal Care Committee at National Institute for Physiological Sciences (approval ID 17A18). These procedures were performed in compliance with the institutional guidelines. Mice were housed in a temperature-controlled room at 22 °C with a 12 h light/dark cycle. Fresh water and rodent diet were available at all times.

### Generation of *miR-124-1*^+/−^ C57BL/6 N strain mice

We backcrossed *miR-124-1*^+/−^ *129S6/SvEvTac* mice^46^ with C57BL/6 N strain mice, using the speed congenic method (Central Institute for Experimental Animals, Japan)^47^, and generated *miR-124-1*^+/−^ C57BL/6 N mice. We confirmed that over 99% of microsatellite DNA markers are from the C57BL/6 N genetic background.

### Plasmid construction

The *Drd2* 3′ UTR or *Nr3c1* 3′ UTR fragment was amplified by PCR using KOD Plus (Toyobo), and cloned into pCR-Blunt vector (Invitrogen). The primer sequences are as follows: for *Drd2* 3′ UTR, forward, 5′-TGAGCTCGACCAGTGTTGGAGCTGAAGT TG-3′ and reverse, 5′-TCTCGAGAGACCCCTCCAAGCTGCAGCTTC-3′; *Nr3c1* 3′ UTR, forward, 5′-CTTGCTAGCCTGCCTTACTAAGAAAGGCTGCCTTAAAG-3′ and reverse, 5′-GAAGTCGACGAAAAACGAGCAAGCATAGTTCACTG-3′. Mutations in the seed match region were introduced by PCR. The primer sequences are as follows: *Drd2* 3′ UTRmut, 5′-GCAAAGTGAGGAGGCTGTGGATGC-3′ and 5′-GAGGCACTGGCAGAGAAGAGACT-3′; *Nr3c1* 3′ UTRmut, 5′-CTTGCTAGCCT GCCTTACTAAGAAAGGCACGGAATTAGAAAGTTG-3′, 5′-CACGGAATTATA GCTATTACTGTCTGG-3′, and 5′-AACTTCCCTTTTCTGATATACACGTGT-3′. The fragments of native and mutated *Drd2* 3′ UTR or *Nr3c1* 3′ UTR were ligated with pmirGLO vector (Promega) to generate pmirGLO-Drd2-3′UTR, pmirGLO-Drd2-3′UTRmut, pmirGLO-Nr3c1-3′UTR, and pmirGLO-Nr3c1-3′UTRmut. To generate AAV-DsRed or AAV-DsRed-miR-124 construct, the fragment of *CAG-DsRed* or *CAG-DsRed-miR-124-2* derived from RIP and RIP-miR-124-2 vectors (a gift from Tom Maniatis, Harvard University) was inserted into pAAV-IRES-hrGFP vector (Agilent technologies). For the production of AAV-shDrd2 and AAV-shControl, a target sequence for *Drd2* (shDrd2)^31^ or shControl^48^ was subcloned into pBAsi-mU6 vector (Takara). The *mU6-shControl* or *mU6-shDrd2* fragment was inserted into the pAAV-CAG-AcGFP vector. For the production of AAV-Drd2, full-length cDNA fragment of mouse *Drd2L* (NCBI #NM_010077) was amplified by PCR using mouse brain cDNA. The primer sequences are 5′-GAATTCGCCACCATGGATCC ACTGAACCTGTCCTGGTAC-3′ and 5′-TCTCGAGCTCAGCAGTGCAGGATCTTC ATGAAG-3′. The fragment was ligated with pAAV-CBh vector, in which the CBh promoter fragment^49^ was inserted into pAAV-CAG-mCherry^50^. To generate pAAV-EGFP, the EGFP fragment from pEGFP-N1 (Clontech) was cloned into pAAV-IRES-hrGFP vector (Agilent technologies).

### Immunofluorescent staining of brain sections

For immunohistochemistry, 30-μm brain sections were washed twice in PBS, permeabilized with 0.1% Triton X-100 in PBS, and then incubated with PBS containing 4% donkey serum for 1 h for blocking. The samples were incubated with a primary antibody at 4 °C overnight. After PBS-washing, these samples were incubated with fluorescent-labeled secondary antibodies at room temperature for 2 h. The specimens were observed under a laser confocal microscope (LSM700, Carl Zeiss). Hoechst (Sigma) was used for nuclear staining. Primary antibodies are as follows: rat anti-GFP (Nacalai, 04404-26, 1:1000) and rabbit anti-DsRed (Clontech, 632496, 1: 1000) antibodies. We used Cy3-conjugated (Jackson ImmunoResearch Laboratories, 1:500) and Alexa Fluor 488-conjugated (Sigma, 1:500) secondary antibodies.

### High-throughput mRNA sequencing (RNA-seq) analysis

Total RNAs from the mouse PFC were extracted using Trizol (Invitrogen). Library preparation was performed using 1 μg of total RNA and NEBNext Ultra Directional RNA Library Prep Kit for Illumina (New England Biolabs). RNA-seq was done with an Illumina HiSeq 1500 for 51 bp single-end. Approximately 40 M reads were obtained in each sample. The reads were mapped against mouse reference sequence. TIGAR2 was run with default settings. The expression level of each gene was quantified as FPKM (fragments per kilobase of exon per million mapped fragments).

### qRT-PCR

Total RNA was extracted using Trizol reagent (Invitrogen), and reverse transcribed into cDNA using SuperScript II reverse transcriptase (Invitrogen) with random hexamers and Oligo dT (Invitrogen). Quantitative PCR was performed using a SYBR GreenER qPCR SuperMix Universal (Invitrogen) and Thermal Cycler Dice Real Time System Single MRQ TP870 (Takara) according to the manufacturer’s instructions. Quantification was carried out by Thermal Cycler Dice Real Time System software version 2.0 (Takara). Nucleotide sequences of primers are as follows: for *Nr3c1*, forward, 5′-CATTTGCCCTGGGTTGGAGATCA-3′ and reverse, 5′-CATGCAGGGTAGAGT CATTCTCTG-3′; *Rpl4*, forward, 5′-GATATGCCATCTGTTCTGCCCT-3′ and reverse, 5′-CTTGCCAGCTCTCATTCTCTGA-3′. To quantify *Drd2* expression level, we used the TaqMan probe (Applied Biosystems) and StepOnePlus Real-Time PCR System (Applied Biosystems). To detect mature *miR-124*, total RNA isolated using the Trizol reagent was reverse transcribed to cDNA using the TaqMan reverse transcription reagent kit (Applied Biosystems) according to the manufacturer’s protocol. qRT-PCR was performed using TaqMan Universal PCR Master Mix (Applied Biosystems) and specific TaqMan mature-miRNA Assays for *miR-124* (Applied Biosystems).

### Animal behavior assays

#### Locomotor activity

Spontaneous locomotor activity was measured as described previously^51^. Briefly, each mouse was placed at the center of open field box (W × D × H = 40 × 40 × 27 cm), and the total distance traveled and the time spent in the center area (W × D = 20 × 20 cm) were scored for 60 min using EthoVision software (Noldus). To examine methamphetamine-induced hyperactivity, mice received one intraperitoneal injection of methamphetamine (1 mg/kg) after 60 min of habituation in open field box.

#### Rotarod

Rotarod was performed as described previously^51^. Male mice were tested on an accelerating rotarod (4–50 rpm in 5 min per test), 3 tests a day (30-min break in-between), around the same time of the day over 3 consecutive days, and the latency to fall was scored.

#### Elevated plus maze

Elevated plus maze was performed as described previously^52^. The plus maze consisted of a plus-shaped apparatus with two open and two closed arms, each with an open roof, elevated 40 cm from the floor. Male mice were put into center of the plus maze and its free movement was recorded for 10 min using EthoVision software.

#### PPI

The startle response and PPI were measured using a startle reflex measurement system (SR-LAB) as previously described^53^ with minor modifications. The test session began by placing a male mouse in a plastic cylinder and leaving it undisturbed for 30 min. The background white noise level in the chamber was 70 dB. A prepulse-pulse trial started with a 50-ms null period, followed by a 20-ms prepulse white noise (74, 78, 82, 86, or 90 dB). After a 100-ms delay, the startle stimulus (40-ms, 120 dB white noise) was presented, followed by a 290-ms recording time. The total duration of each trial was 500 ms. A test session consisted of six trial types (pulse-only trial, and five types of prepulse-pulse trial). Six blocks of the six trial types were presented in a pseudorandomized order such that each trial type was presented once within a block. The formula 100 − ((Response on acoustic prepulse-pulse stimulus trials/Startle response on pulse-only trials) × 100) was used to calculate %PPI.

#### Forced swim test

Each mouse was placed in a transparent glass cylinder (8 cm in diameter × 20 cm high), containing water at 22-23 °C to a depth of 15 cm, and forced to swim for 10 min. The duration of immobility was measured using digital counters with infrared sensors^54^.

#### Tail suspension test

Mice were suspended upside-down by the tail for 10 min. The session was recorded by a video camera and the duration of immobility was measured.

#### Social interaction

Social interaction was performed as described previously^55^. Two mice of identical genotypes previously housed in different cages, were placed into an open field together and allowed to explore freely for 10 min. The behavior was recorded by a CCD camera and automatically analyzed by EthoVision software. The total duration of contacts, the number of contacts, and total distance traveled were measured. Contacts were defined as follows: if the nose of either of the two mice got close to the nose, body or hip of another mouse within 2-3, 5.5–6.5, or 2-3 cm, respectively, the behavior was considered contact.

#### Y-maze

Y-maze was performed as described previously^55^. Each mouse was placed in the center of the symmetrical Y maze and was allowed to explore freely for 10 min. The sequence and total number of arms entered was recorded, using EthoVision software. The sequence triads, in which all three arms were represented (ABC, ACB, BAC, BCA, CAB, and CBA), were calculated as successful alternations to evaluate the normal cognition and working memory of the last arm entered. The percentage of spontaneous alternation is as follows. A number of triads containing entries into all three arms/maximum possible alternations (the total number of arms entered − 2) × 100.

### Slice preparation for electrophysiology

*miR-124-1* heterozygote mice and their littermate control mice of either sex at postnatal 32–35 days were deeply anesthetized with isoflurane and sodium pentobarbital (50 mg/kg, i.p.), and perfused transcardially with ice-cold normal artificial cerebrospinal fluid (ACSF) containing the following: 126 mM NaCl, 3 mM KCl, 1.3 mM MgSO_4_, 2.4 mM CaCl_2_, 1.2 mM NaH_2_PO_4_, 26 mM NaHCO_3_, and 10 mM glucose, saturated with 95% O_2_ and 5% CO_2_. The brains were removed from the mice, and coronal slices (300-μm thick) were prepared from the PFC using a vibrating microslicer (VT1200S; Leica) and recovered in an interface chamber at 33 °C for 1 hour. The slices were then maintained in a submerged chamber at room temperature.

### Electrophysiology

Prefrontal cortical slices containing the prelimbic region were transferred into a submerged-type chamber perfused with normal ACSF. An infrared differential interference contrast video microscopy with an X40, 0.8NA water immersion lens (BX-50WI, Olympus) was used to visualize and target layer 5 pyramidal neurons for whole-cell recordings. Patch pipettes (5–7 MΩ) were filled with a solution containing the following: 130 mM Cs-gluconate, 8 mM CsCl, 1 mM MgCl_2_, 0.6 mM EGTA, 10 mM HEPES, 3 mM MgATP, 0.5 mM Na_2_GTP, 10 mM Na-phosphocreatine, and 0.2% biocytin (pH 7.3 adjusted with CsOH). The membrane potential of the recorded cells was held at the reversal potential of inhibitory postsynaptic currents (IPSCs, -70 mV) or excitatory postsynaptic currents (EPSCs, 0 mV) for EPSC and IPSC recordings, respectively. We did not use series resistance compensation and selected cells with a series resistance < 25 MΩ for analysis. Electrical paired-pulse stimulation was applied at 0.2 Hz through a glass pipette placed in layer 2 just below layer 1, and whole-cell recordings were obtained using patch pipettes located 65 ± 9.0 μm (mean ± SEM) lateral to the stimulating electrode. The interval of the paired-pulse stimulation was 50 and 100 ms for EPSC and IPSC recordings, respectively. To examine the relationship between stimulus intensity and response amplitude, we used stimulus currents that evoked the minimum (3–7 μA), intermediate, and maximum (10–140 μA) EPSC amplitudes. The current intensities used for evoking IPSCs were the same as those employed for evoking EPSCs in each cell. Quinpirole at 5 μM was added to the bath solution and the effect was assessed 10 min after the application. All of the whole-cell recordings were conducted using a Multiclamp 700B amplifier, and data were analyzed using pClamp9 software (Molecular Devices). After whole-cell recording, the slices were fixed with 4% paraformaldehyde in 0.1 M phosphate buffer (PB, pH 7.4) overnight. To visualize the recorded neurons, the slices were incubated with streptavidin conjugated to Alexa 488 (1:1000; Life Technologies) in 25 mM PBS containing 0.1% Triton X-100 overnight at room temperature. Images of slices were obtained using an A1R confocal microscope (Nikon) with a 40x objective.

### AAV production

AAV was produced by triple transfection of an AAV vector plasmid, an adenovirus helper plasmid, and an AAV helper plasmid (pAAV2/9) into AAV-293 cells by the calcium phosphate method. The cells were harvested at 72 h after transfection, and lysed by four freeze-and-thaw cycles. The supernatant was collected by centrifugation, and treated with benzonase nuclease (Novagen) for 10 min at 45 °C to eliminate cellular DNA/RNA and excess plasmid DNAs. The viruses were purified by iodixanol step gradient. The gradient was formed in Ultra-Clear centrifuge tubes (14 × 95 mm, Beckman) by first adding 1.59 ml of 54% iodixanol (Axis-Shield) in PBS-MK buffer (1 × PBS, 1 mM MgCl_2_, and 25 mM KCl) and then overlaying 1.59 ml of 40% iodixanol in PBS-MK buffer, 2.12 ml of 25% iodixanol in PBS-MK buffer containing phenol red, and 3.18 ml of 15% iodixanol in PBS-MK buffer containing 1 M NaCl. Finally, the lysate was applied on top of the gradient. The tubes centrifuged for 3 h at 40,000 rpm at 18 °C in a SW40Ti rotor. The 54–40% fraction containing virus was collected using an 18-gauge needle. The fraction was concentrated using an Amicon Ultra Centrifugal Filter Ultracel-100 K (Millipore). A titer of each AAV (in vector genomes (VG)/mL) was determined by qPCR using SYBR Green ER Q-PCR Super Mix (Invitrogen) and Thermal Cycler Dice Real Time System Single MRQ TP870 (Takara). The primers used for AAV titrations are as follows: for *DsRed*, forward, 5′-ACAAGGTGAAGTTCATCGGCGTGA-3′ and reverse, 5′-AGCTTGGCG TCCACGTAGTAGTAG-3′; *EGFP*, forward, 5′-GAAGGGCATCGACTTCAAGGAG GA-3′ and reverse, 5′-CTTGATGCCGTTCTTCTGCTTGTC-3′; *CBh* promoter, forward, 5′-TCCATTGACGTCAATGGGTGGAGT-3′ and reverse, 5′-CATTGACG TCAATAGGGGGCGTAC-3′.

### Stereotaxic viral injection

AAVs were injected into the PFC of WT and/or *miR-124-1*^+/−^ mice (for PFC, +1.69 mm posterior to the bregma, 0.3 mm lateral from the midline, −1.5 mm depth from the dura). The PPI of injected mice were measured 2 wks after the injection. Coronal sections of the PFC were prepared and immunostained with an anti-GFP or anti-DsRed antibody, followed by the Alexa Fluor 488-conjugated or Cy3-conjugated secondary antibody. Immunofluorescence was detected with a laser confocal microscope (LSM700, Carl Zeiss).

### Luciferase reporter assay

We transfected 0.1 μg of the luciferase reporter vectors (Drd2-3′UTR, Drd2-3′UTRmut, Nr3c1-3′UTR, or Nr3c1-3′UTRmut) and 1.1 μg of the *miR-124* expression vector (pBAsi-mU6-shControl or pBAsi-mU6-pre-miR-124-1)^2^ into HEK293 cells in a 12-well plate using the calcium phosphate method. A β-galactosidase expression vector (β-SV; Promega) was cotransfected for normalization of transfection efficiency. After transfection, the cells were incubated for 48 h and lysed with Reporter Lysis Buffer (Promega). The luminescence signal was detected using a GloMax Multi+ detection system (Promega).

### Electroretinogram recordings

The method of recording the electroretinograms (ERGs) was performed as described previously with some modifications^56^. In brief, mice were dark adapted for more than 4 h, and then anesthetized with an intraperitoneal injection of 100 mg/kg ketamine and 10 mg/kg xylazine. ERG responses were measured using the PuREC system with LED LS-100 (Mayo Corporation). After mice were light adapted for 10 min, the photopic ERGs were recorded on a rod-suppressing white background of 1.3 log cd sm^−2^. Four levels of stimulus intensities ranging from −0.5 to 1 log cd sm^−2^ were used for the photopic ERG recordings. Sixteen responses were averaged for photopic recordings.

### Statistical analysis

Statistical analyses were performed using GraphPad Prism version 6.04 (GraphPad Software). Single comparisons were performed using paired or unpaired Student’s *t* test, and multiple comparisons were performed using one-way ANOVA with *post hoc* Tukey–Kramer test. The statistical significance of experiments involving three or more groups and two or more treatments was assessed by two-way ANOVA with *post hoc* Tukey–Kramer or Bonferroni’s test. Data are reported as median (center line), ±1.5 interquartile range (box), minimal and maximal values (whiskers), as mean ± SEM or as mean ± SD. The analyzed number of samples is indicated in the figure legends. Asterisks indicate significance values as follows: *p < 0.05, **p < 0.01, ***p < 0.001 and ****p < 0.0001.

## Supplementary information

FigS1+S2
