# Supplementary material for: miR-124 dosage regulates prefrontal cortex function by dopaminergic modulation
Source: Sci Rep. 2019 Mar 5;9:3445. doi: 10.1038/s41598-019-38910-2 (PMC6401137; doi:10.1038/s41598-019-38910-2)
Supplement: Supplementary file 1 — FigS1+S2 [file 41598_2019_38910_MOESM1_ESM.pdf]

miR-124 dosage regulates prefrontal cortex function by dopaminergic modulation

Takashi Kozuka[1,2,#], Yoshihiro Omori[1,2,#], Satoshi Watanabe[1,2], Etsuko Tarusawa[3], Haruka Yamamoto[1], Taro Chaya[1,2], Mayu Furuhashi[1], Makiko Morita[4], Tetsuya Sato[5], Shinichi Hirose[7], Yasuyuki Ohkawa[6], Yumiko Yoshimura[3], Takatoshi Hikida[4,8], Takahisa Furukawa[1,2,*]

[1]Laboratory for Molecular and Developmental Biology, Institute for Protein Research, Osaka University, 3-2 Yamadaoka, Suita, Osaka, 565-0871, Japan.

[2]JST, CREST, 3-2 Yamadaoka, Suita, Osaka, 565-0871, Japan.

[3]Division of Visual Information Processing, National Institute for Physiological Sciences, National Institutes of Natural Sciences, 38 Nishigonaka Myodaiji, Okazaki 444-8585, Japan.

[4]Medical Innovation Center, Kyoto University Graduate School of Medicine, 53 Shogoin-Kawahara-cho, Sakyo-ku, Kyoto 606-8507, Japan

[5]Division of Bioinformatics, Medical Institute of Bioregulation, Kyushu University, 3-1-1 Maidashi, Higashi-ku, Fukuoka 812-8582, Japan.

[6]Division of Transcriptomics, Medical Institute of Bioregulation, Kyushu University, 3-1-1 Maidashi, Higashi-ku, Fukuoka 812-8582, Japan.

[7]Department of Pediatrics, School of Medicine, Fukuoka University, 45-1-7 Nanakuma, Jonan-ku, Fukuoka, 814-0180, Japan.

[8]Laboratory for Advanced Brain Functions, Institute for Protein Research, Osaka University, 3-2 Yamadaoka, Suita, Osaka, 565-0871, Japan.

[#]These authors contributed equally to this work.

[*]Corresponding author**:** Takahisa Furukawa

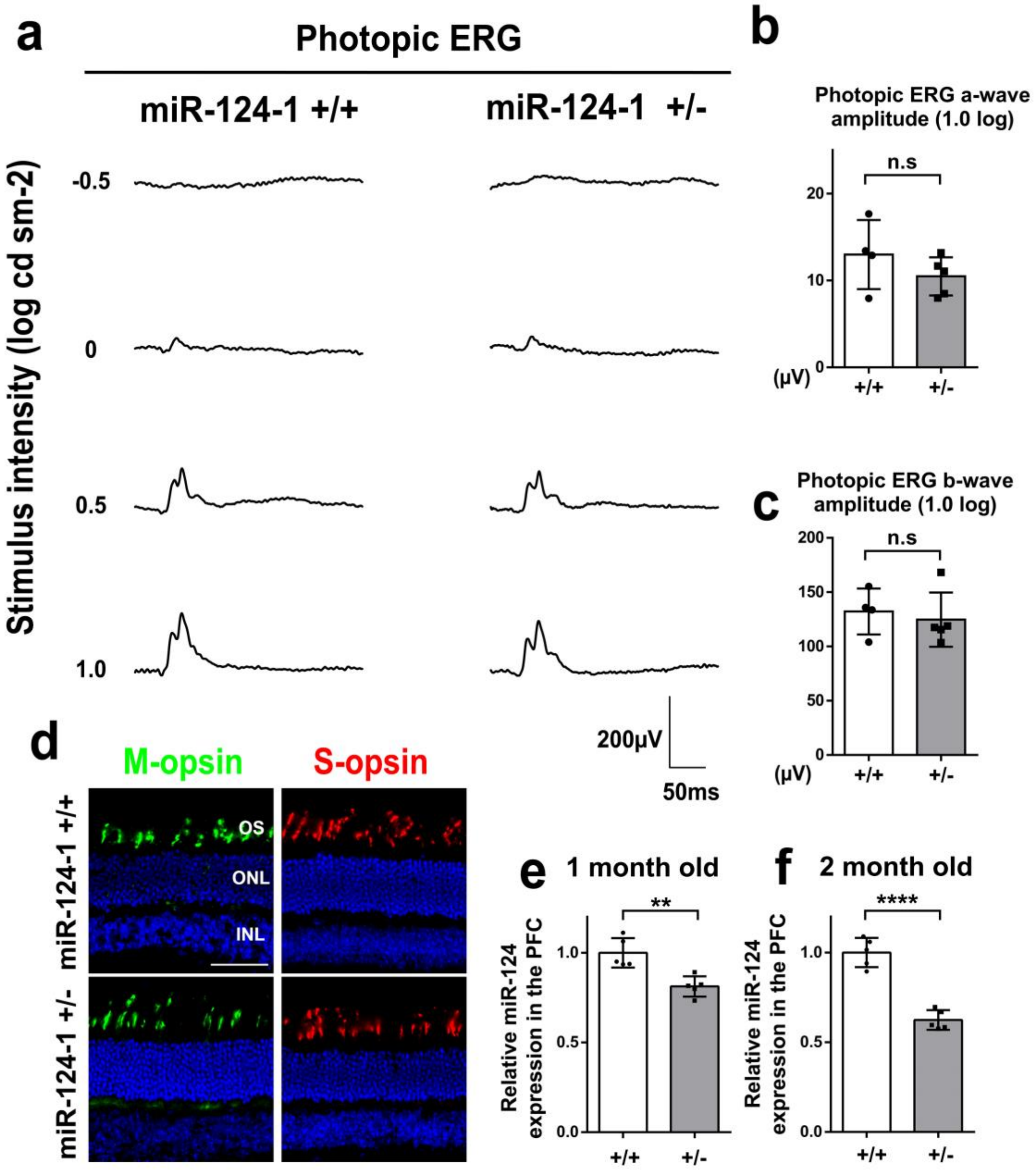

Figure S1. Electrophysiological, histological and molecular analyses of WT and *miR-124-1*$^{+/-}$ mice.

a, Representative light-adapted (photopic) ERGs in WT control and *miR-124-1*$^{+/-}$ mice at 2M elicited by four different stimulus intensities (-0.5, 0, 0.5, and 1.0 log cd sm^{-2}).

b, c, Both a-wave and b-wave amplitudes in *miR-124-1*$^{+/-}$ mice were not significantly altered compared with those in WT mice (WT, n = 4; Het, n = 5; n.s., not significant).

d, The retinal sections of WT and *miR-124-1*$^{+/-}$ mice at 2M were immunostained with the anti-M-opsin (a green-cone outer segment marker, green), and the anti-S-opsin (a blue-cone outer segment marker, red) antibodies. The distribution of the M-opsin and the S-opsin signals was normal in the *miR-124-1*$^{+/-}$ retina. Scale bars, 50 μm. OS, outer segment; ONL, outer nuclear layer; INL, inner nuclear layer.

e, f, The expression level of *miR-124* in the PFC was analyzed using qRT-PCR at 1M (***e***) or 2M (***f***). *miR-124* expression significantly decreased in the *miR-124-1*$^{+/-}$ PFC at 1M and 2M (WT, n = 5; Het, n = 5; ** $p < 0.01$, **** $p < 0.0001$). Error bars represent mean ± SD.

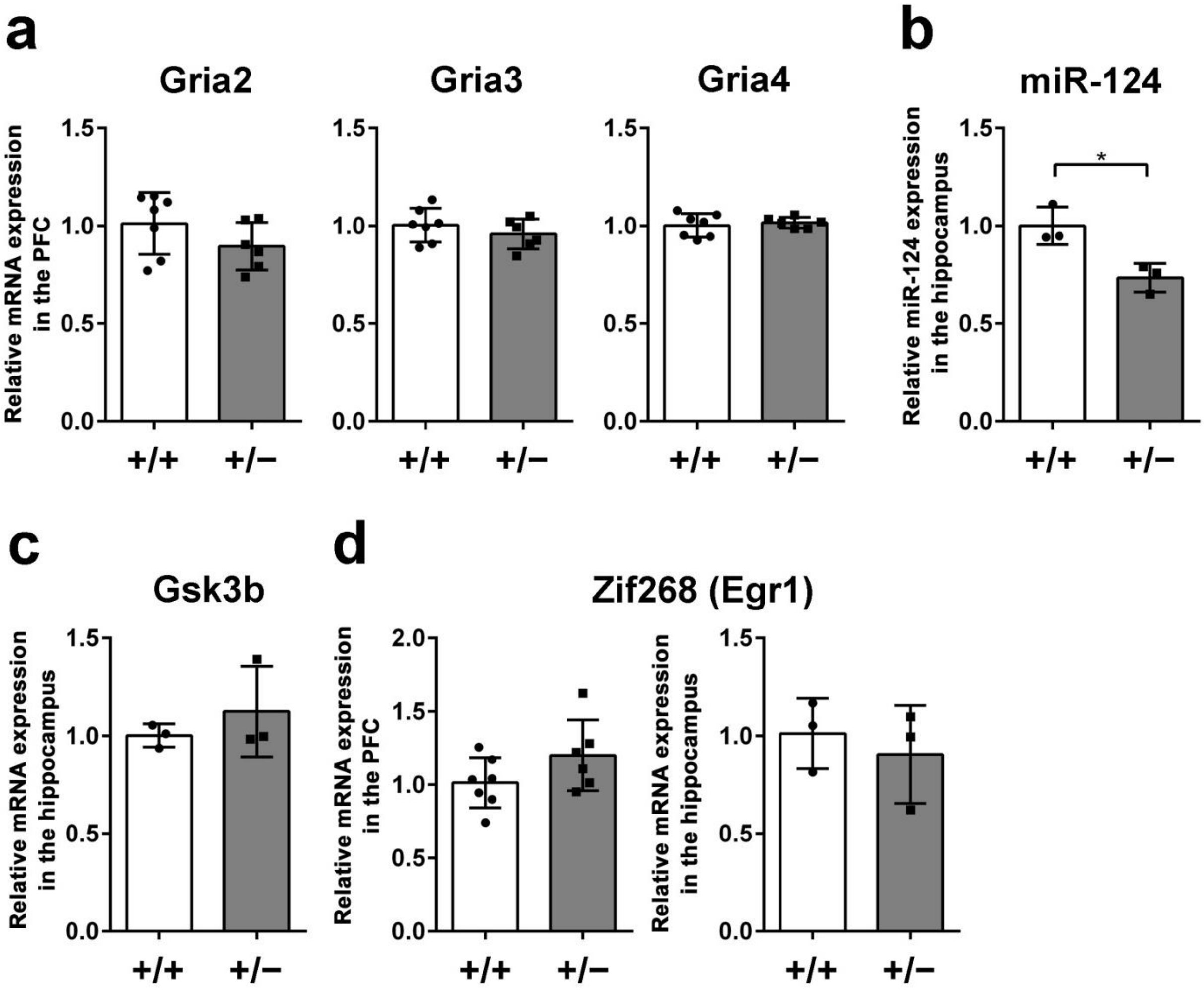

Figure S2. Expression levels of previously reported *miR-124* target genes in the *miR-124-1*$^{+/-}$ PFC and hippocampus.

a, *Gria2, 3* and *4* expressions were not significantly altered between 2M *miR-124-1*$^{+/-}$ and WT PFCs (WT, n = 7; Het, n = 6).

b, Quantification of *miR-124* expression level in the hippocampus. *miR-124* expression significantly decreased in the 2M *miR-124-1*$^{+/-}$ hippocampus (WT, n = 3; Het, n = 3; $^{*}p < 0.05$).

c, *Gsk3b* expression was not significantly affected between the 2M *miR-124-1*$^{+/-}$ and WT hippocampus (WT, n = 3; Het, n = 3).

d, *Zif268 (Egr1)* expression in the PFC (WT, n = 7; Het, n = 6) and hippocampus (WT, n = 3; Het, n = 3) was not significantly altered between the 2M *miR-124-1*$^{+/-}$ and WT mice. Error bars represent ± SD.
